# KIT-5-Assisted Synthesis of Mesoporous SnO_2_ for High-Performance Humidity Sensors with a Swift Response/Recovery Speed

**DOI:** 10.3390/molecules28041754

**Published:** 2023-02-12

**Authors:** Katarina Vojisavljević, Slavica M. Savić, Milica Počuča-Nešić, Aden Hodžić, Manfred Kriechbaum, Vesna Ribić, Aleksander Rečnik, Jelena Vukašinović, Goran Branković, Veljko Djokić

**Affiliations:** 1 Department of Materials Science, Institute for Multidisciplinary Research, University of Belgrade, 11030 Belgrade, Serbia; 2 Center for Sensing Technologies, BioSense Institute, University of Novi Sad, 21102 Novi Sad, Serbia; 3 Center of Excellence for Green Technologies, Institute for Multidisciplinary Research, University of Belgrade, 11030 Belgrade, Serbia; 4 Central European Research Infrastructure Consortium, 34149 Basovizza, Italy; 5 Institute of Inorganic Chemistry, Graz University of Technology, 8010 Graz, Austria; 6 Department for Nanostructured Materials, Jožef Stefan Institute, 1000 Ljubljana, Slovenia; 7 Faculty of Technology and Metallurgy, University of Belgrade, 11000 Belgrade, Serbia; 8 Innovation Center of the Faculty of Technology and Metallurgy, University of Belgrade, 11000 Belgrade, Serbia

**Keywords:** mesoporous silica template, transmission electron microscopy, small- and wide-angle X-ray scattering, tin-dioxide thick-film humidity sensor, X-ray photoelectron spectroscopy, response–recovery behavior

## Abstract

Developing highly efficient semiconductor metal oxide (SMOX) sensors capable of accurate and fast responses to environmental humidity is still a challenging task. In addition to a not so pronounced sensitivity to relative humidity change, most of the SMOXs cannot meet the criteria of real-time humidity sensing due to their long response/recovery time. The way to tackle this problem is to control adsorption/desorption processes, i.e., water-vapor molecular dynamics, over the sensor’s active layer through the powder and pore morphology design. With this in mind, a KIT-5-mediated synthesis was used to achieve mesoporous tin (IV) oxide replica (SnO_2_-R) with controlled pore size and ordering through template inversion and compared with a sol-gel synthesized powder (SnO_2_-SG). Unlike SnO_2_-SG, SnO_2_-R possessed a high specific surface area and quite an open pore structure, similar to the KIT-5, as observed by TEM, BET and SWAXS analyses. According to TEM, SnO_2_-R consisted of fine-grained globular particles and some percent of exaggerated, grown twinned crystals. The distinctive morphology of the SnO_2_-R-based sensor, with its specific pore structure and an increased number of oxygen-related defects associated with the powder preparation process and detected at the sensor surface by XPS analysis, contributed to excellent humidity sensing performances at room temperature, comprised of a low hysteresis error (3.7%), sensitivity of 406.8 kΩ/RH% and swift response/recovery speed (4 s/6 s).

## 1. Introduction

The rise in the average surface temperature on Earth, the greenhouse effect and climate change have increased Earth’s atmospheric water-vapor content, affecting almost all aspects of our lives. Therefore, the necessity for humidity sensor devices that can sense the critical water vapor-content in our environment has grown immensely. They found application in automobile and pharmaceutical/medical industries, agriculture, food and beverage processing, as well as in the control of the living environment in buildings, among other fields [1,2,3].

Such devices are divided into absolute and relative humidity sensors. The latter, including semiconductor ceramics, organic polymer-based and organic/inorganic hybrid sensors (polymer/ceramic), is the most of commercially available and mostly considered for development in laboratory studies [4]. However, superior physicochemical properties in terms of a good mechanical strength, and thermal, physical and chemical stability establish semiconductor metal oxides, SMOXs, as the most promising materials for humidity sensor applications.

Tin (IV) oxide (SnO_2_), a versatile n-type semiconductor with a wide band gap (~3.7 eV) and unique electrical, optical and magnetic characteristics, i.e., with physicochemical properties superior to other SMOXs, has been extensively used in the manufacturing of varistors, solar cells, light emitting diodes [5,6,7], and, particularly, in the production of gas sensors, owing to its high sensitivity to different gas species [8,9]. Good sensitivity to relative humidity, RH, at low temperatures made this SMOX interesting for application as a dampness-sensitive material in RH sensors [10]. Although SnO_2_ has attracted considerable attention as a sensing material, it suffers from some evident drawbacks such as low conductivity, long response/recovery time, and a narrow measuring range [11,12,13]. Especially, its practical application in RH sensors is hindered by a poor response and recovery time.

In attempts to optimize SnO_2_ sensing performance, it is very important to fabricate a nanomaterial with a high specific surface area, and small crystallites with a uniform and well-connected pore structure which can facilitate molecular diffusion and provide abundant active sites for reactions with moisture [14,15,16,17]. For that purpose, various synthesis approaches have been employed, in recent years, to fabricate the SnO_2_ nanomaterial with a high surface area and different morphologies, including the sol-gel method [18,19], hydrothermal reaction [20], spray drying [21], physical co-sputtering deposition [22] and the hard-templating method [16,23]. Hard-templating has appeared as the best method for the synthesis of SMOXs with a high specific surface area and continuous porous nanostructure with adjustable crystallites and pore sizes. This method consists of the synthesis of a highly ordered mesoporous template using the soft templating procedure, thus providing a porous matrix, which is later filled with a precursor for SMOX formation [24,25]. Due to their highly ordered tunable porosity, mesostructured silica materials such as MCM-41, SBA-15, and KIT-6 are most often employed as templates in the synthesis of mesoporous SMOXs via wet hard-templating [24]. The literature reveals that a highly ordered mesoporous SnO_2_ and Ni-doped SnO_2_ replica was synthesized via hard-templating—the incipient wetness [26], vacuum assisted [27], and melt impregnation [28] methods—but with a 3D cubic ordered mesoporous silica KIT-6 used as a template. Juhari et al. showed that mesoporous SnO_2_ derived from SBA-15 [29], SBA-16 and KIT-6 has excellent gas-sensing performances, while Tomer et al. confirmed high RH-sensing performances, including fast response/recovery speed, when the SBA-15-supported Ag-SnO_2_ was used as a sensing layer [23].

Among other mesostructured silica, the KIT-5 lately appeared as an interesting 3D cubic close-packed (ccp) cage-type material. For applications involving selectively tuned diffusion and host–guest interactions with nanostructured SMOX materials, the KIT-5 structure with its interconnected pore channels has shown to be superior to hexagonal silica templates having one-dimensional channels [30]. Even though various highly ordered mesoporous SMOXs have been synthesized using a wet hard-templating approach [31,32], not much attention has been paid to the mesoporous SnO_2_ replica derived from the KIT-5 matrix.

Rajalakshmi et al_._ reported on the direct hydrothermal synthesis of highly ordered SnO_2_-supported KIT-5 hybrids with large specific surface areas [33]. However, an extensive literature search on mesoporous SnO_2_ replicas derived from the KIT-5 matrix using the hard-templating, wet impregnation, method confirmed only one study related to this topic [34]. The gas sensor processed from the SnO_2_ replica showed a strong size-induced enhancement of gas-sensing performance toward acetone and ethanol vapors using dry air as a reference gas [34]. To the best of our knowledge, the mesoporous SnO_2_ derived from the KIT-5 silica template through the wet impregnation process has not been used before as the sensing material for the fabrication of humidity sensors.

Apart from the benefits associated with the mesoporous architecture of SnO_2_, such as small crystallites and interconnected pore channels which can provide abundant sites for interaction with the analyte of interest, the vital gas/humidity sensing parameters such as the sensitivity, linearity, response/recovery speed and hysteresis behavior of the SnO_2_ sensors can be fine-tuned by modifying their composition with noble/transition metals, metal oxides, graphene and/or graphitic carbon nitride, among others [16,21,22,23,35,36,37]. Such modifiers can act as catalysts and increase the concentration of reactive species (oxygen vacancies and other oxygen-like defects) on the surface and/or decrease the activation energy of the surface process, contributing to improved interaction with gas/water molecules. The dopant-induced non-stoichiometry in SnO_2_ can readily affect the resistance and charge transport through the material, making it more or less suitable for gas/humidity-sensing applications [35,38].

Herein, the mesoporous SnO_2_ nanomaterial was prepared via the hard-templating—wet impregnation—method utilizing, for the first time, as a host the mesostructured KIT-5 matrix synthesized by the aging of appropriate reagents in low acidic media and under a static hydrothermal condition at 130 °C, instead of the usually explored treatment at 100 °C. Additionally, mild leaching conditions (1 M NaOH, 40 °C, 6 h) were used to dissolve the silica template. Synthesis parameters defined in this way can significantly affect the quality of the final product (SnO_2_ replica). They can induce changes in the specific surface area, pore concentration and structure ordering, as well as increase the concentration of reactive species on the material’s surface, affecting its interaction with water molecules. From that point of view, this article reveals the nature and quality of the synthesized products, KIT-5, SnO_2_-KIT-5 hybrid and SnO_2_ replica, and defines the potential of SnO_2_ replica to be used as an effective humidity sensing layer. For comparison purposes, the SnO_2_ nanomaterial was also synthesized using a conventional sol-gel method. Thick films were deposited by the doctor blade technique from the pastes based on SnO_2_ powders. The sensor response to the humidity of the thick films, including their adsorption/desorption behavior and response/recovery time, was tested by measuring the change in the complex impedance. To analyze in-depth factors that can affect the surface reactivity to humidity, the XPS analysis of both sensors was performed. Overall investigation elucidates the relationship between morphology, surface phase composition and humidity sensing behavior. At the same time, it explains the possible sensing mechanism and reasons for the improved sensitivity and fast response/recovery time observed in sensors developed from the mesoporous SnO_2_ replica.

## 2. Results and Discussion

### 2.1. Morphological/Structural Characterization of Materials Using TEM, SAXS/WAXS and BRT/BJH Methods

In this study, 3D cage-type mesoporous KIT-5 silica was fabricated using the Ryoo’s procedure. The lyotropic liquid-crystalline phase was formed under a low HCl concentration and matured at 45 °C, allowing the thermodynamic control of the silica-triblock copolymer mesophase self-assembly. According to Ryoo’s investigation, it is expected that hydrothermal treatment at higher temperatures (100 °C ˂ T_HT_ ≤ 130 °C) and at a constant reaction time of 24 h improves pore size distribution and the formation of the larger pores of the KIT-5 template, maintaining its original *ccp* structure [30].

**Figure 1:**
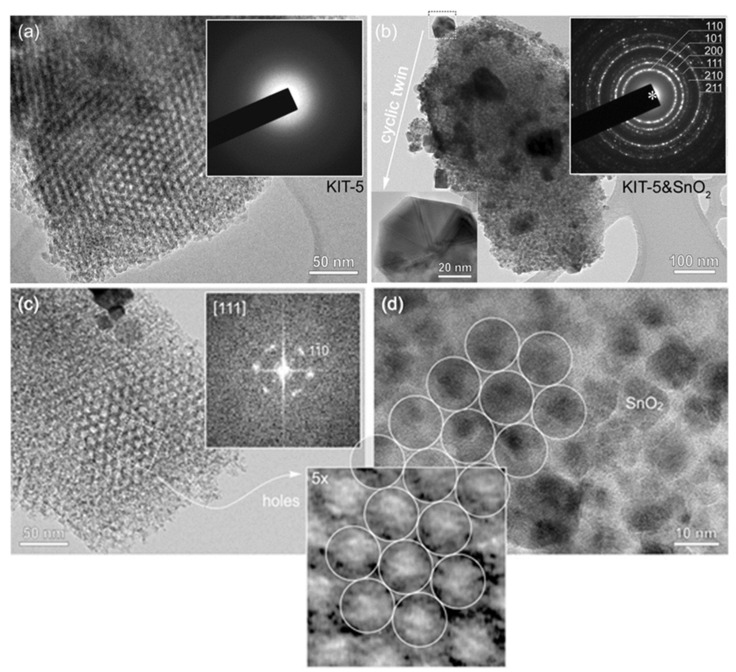
TEM study of (**a**) KIT-5 with pseudohexagonal pore distribution along [111] projection along with the diffraction pattern from the corresponding area (inset); (**b**) KIT-5 matrix with homogeneously distributed fine-grained SnO_2_ and few exaggerated, grown cassiterite crystals. Insets: SAED ring-pattern of cassiterite (up); the cassiterite cyclic twin (down); (**c**) KIT-5 pore distribution within SnO_2_-KIT-5, [111] projection. The FFT pattern of corresponding area is shown as inset; (**d**) TEM image of SnO_2_ nanoparticles incorporated in KIT-5, following a honeycomb arrangement of pores. The inset across (**c**,**d**) shows 5x-magnified image of pores from [111]-oriented KIT-5 from (**c**) for comparison of SnO_2_ grain sizes in (**d**).

To prove mesostructure ordering, KIT-5, SnO_2_-KIT-5, as well as the SnO_2_-R after the removal of the KIT-5 template were characterized by TEM. The TEM image of KIT-5 (Figure 1a) shows a highly ordered mesoporous structure and empty pores with a honeycomb arrangement along the <111> axes. Electron diffraction recorded from KIT-5 flakes shows a homogeneous background with no diffraction rings indicative of amorphous materials. TEM analysis of the SnO_2_-KIT-5 cluster, shown in Figure 1b, reveals that nucleation of SnO_2_ nanocrystals takes place in the interior of the KIT-5 matrix, but also on its surface. In most of the analyzed KIT-5 clusters, pores are homogeneously filled by SnO_2_ which undergoes normal crystal growth following the matrix structure. These globular SnO_2_ particles do not exceed the size of 10 nm. However, in some examples, when all the pores of KIT-5 are occupied (Figure 1b), a few larger SnO_2_ crystals (diameter 50–100 nm) appear on the surface of KIT-5 particles. HRTEM analysis of these exaggerated, grown SnO_2_ crystals reveals that they are in fact all cyclic twins of cassiterite. One such cyclic twin, tilted edge-on (inset Figure 1b), shows that they are crystallographic eightlings, where up to eight individuals are twinned by {101} planes around the common <111> axis [39]. The trigger of twinning is not known. Like in the case of undoped and doped SnO_2_ and ZnO ceramic samples, some nonstoichiometry-related lattice strains or topotaxial replacement during unknown precursor phases may be the possible reasons for twinning and exaggerated growth [40,41]. However, a detailed analysis must be performed to give any precise explanation. A further interesting observation is the increase in SnO_2_ cell parameters, measured from SAED patterns of SnO_2_ in the SnO_2_-KIT-5 cluster. The intensities and distribution of diffraction rings (inset in Figure 1b) correspond to a cassiterite structure with slightly higher *d* values in comparison to those of SnO_2_-R (Table S1). This can be indicative of the formation of the SnO_2_-KIT-5 complex and nano-sized particle constraints. TEM analysis of KIT-5 mesostructure symmetry within SnO_2_-KIT-5 reveals that the mesopores are highly ordered (Figure 1c,d, Figure S1). The mesostructure ordering was further studied in three low-index projections: [001], $\left[1\bar{1}0\right]$ and [111] (Figure 1c and Figure S1). As estimated from the TEM images and the corresponding fast Fourier transform (FFT) patterns from ordered regions, the apparent *d* values are: *d*_220_ = 6.0 nm, *d*_200_ = 8.5 nm, *d*_111_ = 9.8 nm and *d*_110_ = 12.0 nm. Lattice parameter *a* of the *ccp* cell, determined from the *d* values, is 17.0 nm. Pore distribution confirms a pseudocubic mesostructure which can be described by the $Fm\bar{3}m$ symmetry group. The average pore diameter and wall thickness, measured at the full width at half maxima (FWHM) along the [110] direction from Figure 1c, recorded close to the Gauss focus, are around 6.4 nm (pores) and 5.4 nm (walls). It must be mentioned that in TEM, these values can strongly vary due to the delocalization of the contrast as a function of particle thickness and defocus and can be taken only as rough approximates [42]. Figure 1d shows a honeycomb arrangement of SnO_2_ nanoparticles in SnO_2_-KIT-5. Detailed analysis shows that the size of the particles (~10 nm) and their apparent pseudohexagonal distribution matches that of the KIT-5 pores viewed in the [111] projection, Figure 1c. The periodic arrangement of SnO_2_ nanoparticles in KIT-5 replicates the mesostructure of KIT-5 pores and confirms that SnO_2_ particles nucleated within the KIT-5 framework. It can be confirmed that KIT-5 affects and dictates the nucleation of, and growth in, SnO_2_ nanoparticles.

**Figure 2:**
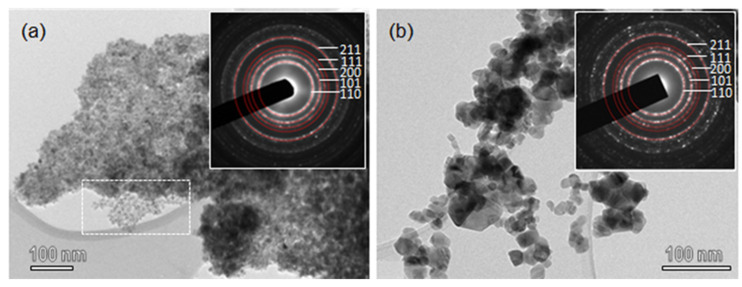
TEM images of (**a**) SnO_2_-R and (**b**) SnO_2_-SG. The insets in (**a**,**b**) show SAED patterns from the corresponding areas of SnO_2_-R and SnO_2_-SG samples.

TEM images of SnO_2_-R and SnO_2_-SG are shown in Figure 2. SnO_2_-R powder consists of small particles uniform in size and shape. It can be seen that after the removal of silica (Figure 2a), SnO_2_ nanoparticles replicate the structure of KIT-5, but with a lower degree of ordering compared to KIT-5 and SnO_2_-KIT-5. The area in Figure 2a, marked with a rectangle, is an example of the highly ordered SnO_2_, which follows the structure of the KIT-5 matrix. The SnO_2_-R composition was confirmed using EDXS analysis (Figure S2), and the content of Si is minimal, less than 0.5%. This result confirms that mild leaching conditions accompanied with slow calcination allowed not only the preservation of the KIT-5 structure, but almost the complete removal of silica from the SnO_2_-R. Compared to SnO_2_-R, the TEM image of SnO_2_-SG (Figure 2b) shows coarse crystallinity with particles of diverse shapes and sizes, ranging from 10–50 nm. Well-defined rings observed in SAED patterns (inset in Figure 2a,b) were indexed as the (110), (101), (200), (111) and (211) lattice planes of a *P*42/*mnm* cassiterite; the d-spacing values are in accordance with JCPDF 01 077-0452.

**Figure 3:**
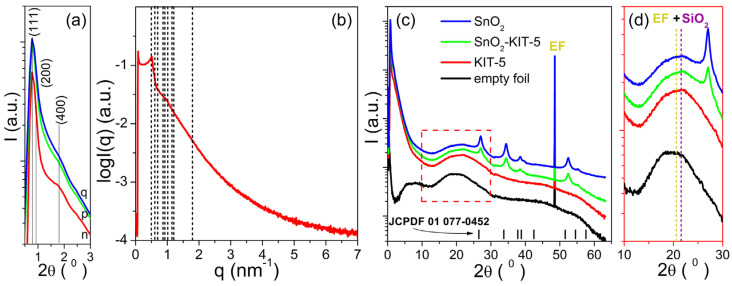
SAXS measurements: (**a**) comparing all samples with y log scale; (**b**) fitting the KIT-5 $Fm\bar{3}m$ structure with the lattice parameter *a* = 20.14 nm; (**c**) simultaneous SWAXS patterns of all samples; (**d**) an enlarged part of the WAXS patterns between 10–30° (2θ), where the positions of the peaks at 20° (2θ) from the empty foil and at 22° (2θ) from the amorphous SiO_2_ are marked with dashed lines. Samples are denoted as: n—KIT-5, p—SnO_2_-KIT-5 and q—SnO_2_-R. The tick marks in (**c**) correspond to the peak positions of the *P*42/*mnm* cassiterite.

The SAXS patterns of the KIT-5, SnO_2_-KIT-5, SnO_2_-R and empty foil are shown in Figure 3a. The simulated pattern of the KIT-5 (see Figure 3b) reveals one distinct peak observed at 0.77° and two shoulders positioned at 0.88° and 1.31° (2θ) indexed to (111), (200) and (220) planes characteristic for the $Fm\bar{3}m$ structure of the KIT-5 material [30,33]. The hump detected at 1.79° (2θ) is related to the (400) plane of the $Fm\bar{3}m$ structure, but its high intensity could be indicative of the self-assembly of building blocks into a mixed cubic structure. The *d* spacing (*d*_111_ = 2π/*q*) and the unit cell parameter ($a_{o}=d_{111}\sqrt{3}$) calculated based on the strongest (111) peak of the KIT-5 were found to be 11.63 and 20.14 nm, respectively. The main structure of the KIT-5, still visible in SnO_2_-KIT-5, is less pronounced. That can be related to the incorporation of the SnO_2_ into the KIT-5 framework and even the formation of nanosized SnO_2_ species which are implemented in the silica framework. All these lower the starting order degree of KIT-5, and this causes the observed decrease in reflections intensities in the SnO_2_-KIT-5 pattern, Figure 3a. Even though the leaching process along with the thermal treatment has a negative influence on reflection intensities, the SAXS pattern of SnO_2_-R confirms the partial preservation of the mesoporous structure, Figure 3a. The SWAXS patterns of the KIT-5, SnO_2_-KIT-5 and SnO_2_-R along with the empty foil are shown in Figure 3c. According to the spectra, the only sample without signals from the crystalline phase is the pure KIT-5, which remains amorphous in the measured WAXS region. WAXS patterns of SnO_2_-KIT-5 and SnO_2_-R (Figure 3c) display well-resolved diffraction peaks which can be indexed to a tetragonal structure of SnO_2_ (JCPDF 01 077-0452). They also display the broad peak centered at 22° (2θ) characteristic for the amorphous silica walls mixed with the signal from the empty foil (Figure 3d). Due to these mixed signals, it is difficult to talk about the purity of SnO_2_-R based only on the WAXS pattern. However, a small amount of silica was previously confirmed by EDXS analysis (Figure S2). The average crystallite size of the SnO_2_-R particles calculated by applying the Debye–Scherer equation (Equation (S1)) appeared to be 10 nm. For comparison, the average crystallite size of the SnO_2_-SG particles was also calculated from the corresponding pattern (Figure S3), and it was found to be approximately 4 times larger than that observed in the SnO_2_-R powder.

A deeper insight into products’ mesoporous structure can be obtained by porosimetry, such as BET analysis, and the analytical modeling of the SAXS data by applying the Guinier–Porod law. In addition, the Guinier–Porod law applied on SAXS data allows extracting the real-space information contained in the scattering curves [43,44].

**Figure 4:**
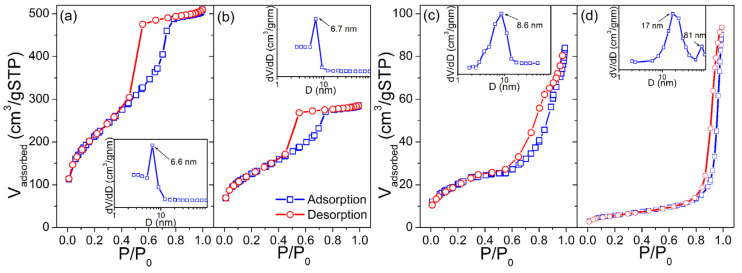
Nitrogen adsorption/desorption isotherms and corresponding pore size distributions (inset) of the (**a**) KIT-5, (**b**) SnO_2_-KIT-5, (**c**) SnO_2_-R and (**d**) SnO_2_-SG powders.

Corresponding N_2_ adsorption/desorption isotherms of KIT-5, SnO_2_-KIT-5, SnO_2_-R and SnO_2_-SG powders and BJH pore size-distribution plots obtained from desorption branches are shown in Figure 4. The specific surface areas, *S*_BET_, of the powders were found to be 760, 442, 66.7 and 20.4 m^2^·g^−1^, respectively. Using the formula for the BET equivalent-particle diameter (Equation (S2), [45]), the primary particle size values were calculated to be 12.9 and 42.3 nm for the SnO_2_-R and SnO_2_-SG, respectively, which are in-agreement with WAXS-XRD/TEM findings. The KIT-5, SnO_2_-KIT-5 and SnO_2_-R, according to the IUPAC classification [46], exhibit type-IV isotherms with H1 hysteresis loops, indicative of well-defined cylindrical pore channels with large uniform cage-type porosity and capillary condensation within uniform pores. According to BJH (see insets in Figure 4a,b), KIT-5 and SnO_2_-KIT-5 display a monomodal narrow pore size distribution with peaks located at 6.6 and 6.7 nm, respectively. The loading of the host KIT-5 matrix with SnO_2_ led to a decrease in the pore volume, *V*_pore_ from 0.71 to 0.39 cm^3^·g^−1^. In the case of SnO_2_-R, the shape of adsorption–desorption isotherms is partially preserved, providing proof of the partial conservation of the mesoporous structure of the host matrix after SnO_2_ loading and silica removal (see Figure 4c). The BJH plot of this sample reveals a pore size distribution slightly broader than in the case of KIT-5 and SnO_2_-KIT-5 with a peak centered at 8.6 nm. In addition, the SnO_2_-SG exhibits the type-IV isotherm with a H3 hysteresis loop, which can be related to capillary condensation within meso- and macropores. The BJH plot confirms broad, non-uniform pore size distribution with maxima around 17 and 81 nm, and *V*_pore_ of 0.12 cm^3^·g^−1^.

Although the BJH plot of SnO_2_-SG revealed that most of the pore diameters are close to 17 nm, it cannot compete with SnO_2_-R. Contrary to SnO_2_-SG, with its poor morphological characteristics, the SnO_2_-R possesses a large *S*_BET_ of 66.7 m^2^·g^−1^, *V*_pore_ of 0.22 cm^3^·g^−1^ and relatively ordered mesoporous structure formed from SnO_2_ particles uniform in size (8–10 nm) and shape, and separated from each other with mesopores that exhibit an almost narrow pore size distribution, with a maximum at 8.6 nm.

As mentioned earlier, analytical modeling of the SAXS data by applying the Guinier–Porod law along with BET analysis can provide more insights regarding the mesoporous structure of analyzed materials. Thus, the Guinier–Porod model was applied on SAXS data. More details on the Guinier–Porod model can be found in Supplementary Materials*, SM*. The radius of gyration, *R*_g_; the SAXS invariants of the first moment of the smeared intensity, Q and K; and surface to volume ratio, *S*/*V* were calculated and are given in Table 1. Starting from the predominantly spherical structure of the SnO_2_-R material, the *R*_g_ was calculated taking into account its sphere-like structure [47]. Note that the *R*_g_ is related to the mean size of the scattering pores in the sample [48]. While the average pore size in the KIT-5 is 6.6 nm, this value increases to 13.6 nm after SnO_2_ loading, as well as to 14.4 nm after silica leaching, indicating the increase in porosity of SnO_2_-KIT-5 and SnO_2_-R samples, respectively. As can be seen from Table 1, there is a good agreement between the *R*_g_ and ${\bar{d}}_{\mathrm{meso}}^{\mathrm{BET}}$ values obtained by SAXS and BET analyses for KIT-5. However, some discrepancies are observed in the case of SnO_2_-KIT-5 and SnO_2_-R samples. The *R*_g_ values of the SnO_2_-KIT-5 and SnO_2_-R are influenced by the templating method, i.e., loading of the host KIT-5 matrix with SnO_2_ and further removal of the silica template. These values indicate the presence of closed pores in the mentioned samples. Such pores are more visible using X-ray in SAXS than by N_2_ adsorption in the BET analysis. It is expected that the *R*_g_ values of SnO_2_-KIT-5 and SnO_2_-R most probably originate from entering the SnO_2_ not only in the mesopores but also into the walls of the KIT-5 matrix [49]. On the other hand, the *S*/*V* rapidly changes from 277 m^2^·cm^−3^ (KIT-5) to 72 m^2^·cm^−3^ (SnO_2_-KIT-5), implying the collapse of KIT-5 mesoporous structure as the result of 10 wt% Sn-loading. Why the *S*/*V* value is much lower in the SnO_2_-KIT-5 can be explained by the fact that the *R*_g_ value of KIT-5 is approximately twice smaller than that of SnO_2_, and that pore channel disintegration occurs when the SnO_2_ enters the KIT-5 pores. In addition, as a result of overloading, out-the-host-SnO_2_ species are observed at the surface of SnO_2_-KIT-5 clusters by TEM (Figure 1b), influencing, at the same time, a decrease in the *S*/*V* ratio in the SnO_2_-KIT-5 sample. Finally, the *S*/*V* ratio of the SnO_2_-R was found to be twice as high as in the case of SnO_2_-KIT-5, indicating that the removal of silica resulted in a sample with sufficiently high porosity to be further used as humidity sensor.

### 2.2. Humidity Sensing Performances of Sensors Based on Mesoporous and Conventionally Processed Tin Oxides

**Figure 5:**
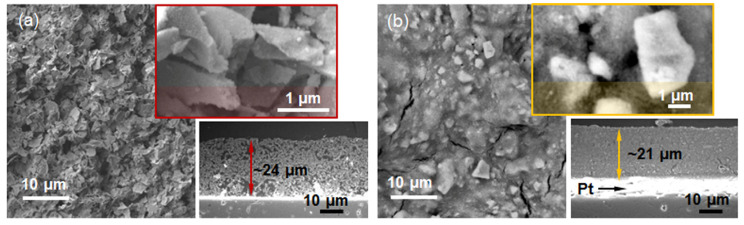
SE micrographs of the thick films: (**a**) TF(SnO_2_-R) and (**b**) TF(SnO_2_-SG), with corresponding enlarged part of their surfaces (up) and cross sections (down).

Inspired by the high specific surface area, relatively ordered porous structure, and uniform distribution of crystallites’ shape and size observed in SnO_2_-R, we explored its potential application in humidity sensors. Following the procedure explained in *Materials and Methods*, Section 3.5, the TF(SnO_2_-R) and, for comparison, the TF(SnO_2_-SG) sensors were processed and their microstructures are presented in Figure 5.

The highly porous TF(SnO_2_-R) with an average sensor thickness of 24 µm consists of ~2 µm plate-like particles (Figure 5a). From the enlarged part (the upper inset in Figure 5a), it can be seen that these plate-like particles are built from small particles of a few tens of nanometers. In contrast to TF(SnO_2_-R), the cross-sectional microstructure and the top surface of 21 µm thick TF(SnO_2_-SG) (Figure 5b) reveal a quite dense and cracked structure built of 2–5 µm sized particles. Such a difference in microstructure could be responsible for the dissimilar functional characteristics of the prepared sensors.

The humidity sensing performance of the TF(SnO_2_-R) and TF(SnO_2_-SG) was evaluated by measuring the complex impedance when switching the humidity between 30 and 90% RH at two operating temperatures (25 and 50 °C). To make it easier to follow, only the results collected at 25 °C are presented in the main text, while those collected at 50 °C are shown in *SM*.

**Figure 6:**
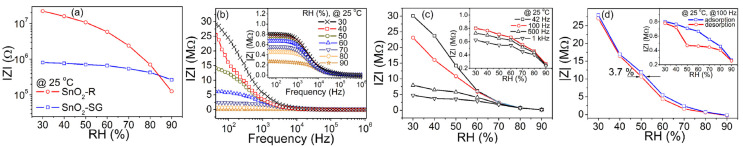
(**a**) The humidity sensing curves of SnO_2_-R and SnO_2_-SG sensors given as the change in |Z| over 30–90% RH; (**b**) the change in |Z| with frequency for the TF(SnO_2_-R) and TF(SnO_2_-SG) (inset) exposed to certain RH values; (**c**) performance of the TF(SnO_2_-R) and TF(SnO_2_-SG) (inset) expressed as the change in |Z| over 30–90% RH at specific frequencies; (**d**) hysteresis behavior of the TF(SnO_2_-R) and TF(SnO_2_-SG) (inset) over 30–90% RH.

Figure 6a shows the change in complex impedance with a relative humidity change for both sensors. The TF(SnO_2_-R) and TF(SnO_2_-SG) exhibit significantly different humidity sensing curves with 2.3 and 0.5 orders of change in the impedance magnitude, respectively. A high specific surface area and still-observable ordered mesopore channels in SnO_2_-R (see Figure 2a) enable specific interaction with water molecules and smooth propagation of charge carriers, resulting in the difference in the impedance magnitude. Figure 6b and the inset in Figure 6b present the frequency dependence of the complex impedance of both sensors, measured in the 30–90% RH range at 25 °C. For the TF(SnO_2_-R) sensor, the measured impedance decreases with an increase in frequency from 42 Hz to 1 MHz at all RH values, but the shape of the measured curves, |*Z*| vs *f*, strongly depends on the RH. As can be seen from Figure 6b, with an increase in RH, the impedance moderately decreases with frequency, especially above 70% RH. In addition, the rise in the RH prominently influences a decrease in complex impedance at the lower frequency range. For example, at 42 Hz the impedance of 29.0 MΩ is measured at 30% RH and it lowers to 158 kΩ at 90% RH. Measured under the same conditions, the TF(SnO_2_-SG) sensor shows an insignificant change in impedance from 802 kΩ at 30% to 264 kΩ at 90% RH, inset in Figure 6b. Figure 6c shows the effect of frequency variation from 42 Hz to 1 kHz in the 30–90% RH range on the performance of the TF(SnO_2_-R) sensor. A reduction in the sensor’s impedance is observed upon RH increase, when a continuous water layer is formed at the sensor’s surface, i.e., when the water molecules become less close with their neighbors. In contrast to low frequencies, at higher frequencies the impedance becomes less dependent on the RH, as can be seen from Figure 6c. Considering both the high change in impedance and relatively linear response over the measured RH range (Figure 6c and an inset in 6c), the frequency of 100 Hz was chosen for further evaluation of sensing performances of the TF(SnO_2_-R) and TF(SnO_2_-SG). The time delay between adsorption and desorption processes, measured at 100 Hz and 25 °C for both sensors, are presented in Figure 6d. Contrary to TF(SnO_2_-SG) (inset in Figure 6d), the adsorption and desorption curves for the TF(SnO_2_-R) nearly overlay each other, showing a small hysteresis error of 3.7% (i.e., 3.2% @ 50 °C; Figure S4c), which represents the good reliability of this sensor. In general, the higher operating temperature, 50 °C, resulted in a similar sensor performance for the TF(SnO_2_-R) to that observed at 25 °C, but with overall lower characteristics (Figure S4).

**Figure 7:**
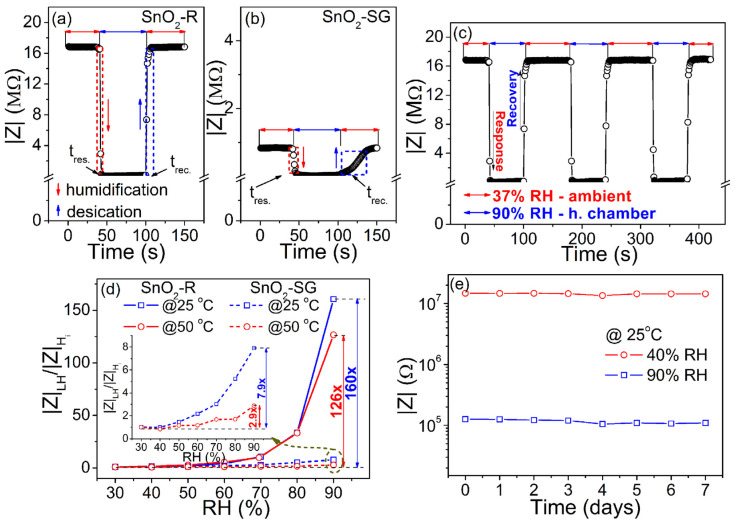
Response/recovery time, measured at 25 °C and at frequency of 100 Hz, for (**a**) the TF(SnO_2_-R) and (**b**) TF(SnO_2_-SG); (**c**) reproducibility of the TF(SnO_2_-R) sensor; (**d**) TF(SnO_2_-R) and TF(SnO_2_-SG) (inset) sensor responses calculated as |*Z*|_LH_/|*Z*|_(H)i_ at frequency of 100 Hz and at temperatures of 25 and 50 °C; (**e**) stability of the TF(SnO_2_-R) sensor measured at 25 °C and 100 Hz.

Following the change in impedance at 100 Hz/25 °C during the humidification/desiccation process, the response/recovery time measured for TF(SnO_2_-R) and TF(SnO_2_-SG) is presented in Figure 7a,b. The TF(SnO_2_-R) sensor shows a swift response in adsorption/desorption processes, with a response time of 4 s and recovery time of 6 s (Figure 7a). However, the same processes for the TF(SnO_2_-SG) sensor are considered to be slow (Figure 7b), since the response/recovery time was found to be 16/40 s. The dynamic response of the TF(SnO_2_-R) was followed by measuring the impedance during the sensor’s switching between the external environment (37% RH) and humid chamber (90% RH) in three reiterated cycles (Figure 7c). These measurements proved that the sensor is highly reversible and possesses excellent reproducibility. Additionally, the sensor responses of both films were followed at 100 Hz and at operating temperatures of 25 and 50 °C, as shown in Figure 7d. Contrary to TF(SnO_2_-SG), the TF(SnO_2_-R) sensor exhibits a significant reduction in impedance at higher relative humidity values (above 60% RH). In fact, the sensor response of TF(SnO_2_-R), measured at 25 °C, reduces 160 times within the 30–90% RH range, while it tended to decrease in a moderate manner (it reduces by 126 times) at 50 °C under the same frequency and RH range. Compared to TF(SnO_2_-SG), the sensor response of TF(SnO_2_-R) is 20 and 43 times larger when measured at 25 and 50 °C, respectively. The sensitivity, *S*, of both sensors was determined using the equation $S=\left(\left|\Delta \left|Z\right|\right|/{\left|Z\right|}_{0}\left(\%\right)\right)/\Delta RH$ [50], (where ${\left|Z\right|}_{0}$ is the impedance of the sensor at 30% RH, |Δ|Z|| is the change in impedance, and ΔRH is the RH change). It was found to be 2.66%/RH% (i.e., 406.8 kΩ/RH%) and 0.04%/RH% (i.e., 8.9 kΩ/RH%), respectively, measured at 25 °C. The investigation of the TF(SnO_2_-R) sensor performance toward 90% and 40% RH (close to the measured external environment humidity of 37% RH) over 1 week (Figure 7e) shows that the response values at both RH% are well-retained, without distinct differences. This reflects the good long-term stability of the TF(SnO_2_-R) sensor and confirms its practical application as a humidity sensor.

**Figure 8:**
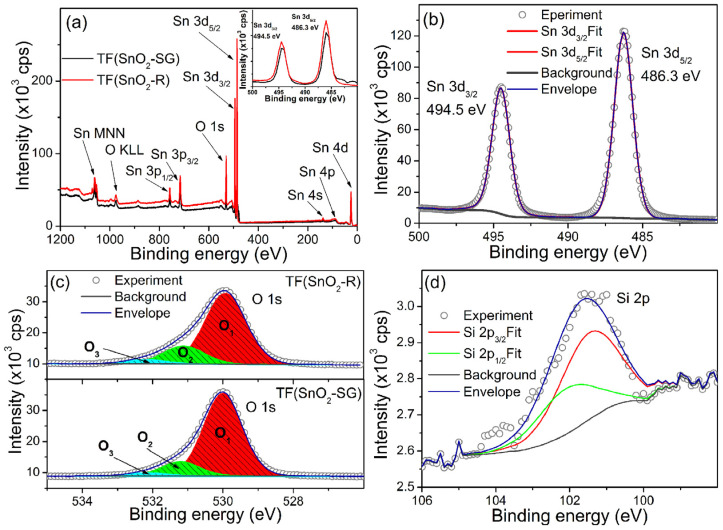
XSP spectra: (**a**) survey spectra of TF(SnO_2_-R) and TF(SnO_2_-SG) with an enlarged section representing Sn 3*d* line in the inset; (**b**) high-resolution spectrum of Sn 3*d* line recorded at TF(SnO_2_-R); (**c**) high-resolution O 1*s* line from TF(SnO_2_-R) and TF(SnO_2_-SG) and (**d**) high-resolution Si 2*p* line observed in TF(SnO_2_-R) spectrum.

To analyze in-depth reasons for its improved sensor response, sensitivity and response/recovery speed, the XPS analysis of TF(SnO_2_-R) was conducted in order to investigate and compare its surface elemental composition with its TF(SnO_2_-SG) counterpart (Figure 8). The survey XPS spectra of both samples are presented in Figure 8a. The Sn and O photoelectron lines including Auger Sn MNN and O KLL lines were clearly observed, identified and marked by the arrows. The high-resolution spectrum of Sn 3*d* (Figure 8b) revealed that Sn 3*d*_5/2_ and Sn 3*d*_3/2_ lines are located at binding energies of ≈486.3 and ≈494.5 eV, respectively. The positions of these lines (Sn 3*d*_5/2_ and Sn 3*d*_3/2_) for both spectra are located at same binding energies (Inset Figure 8a), and their fits consist of a single contribution, indicating that Sn exists only in the Sn^4+^ valence state in both samples. By contrast, a certain difference is observed in the high-resolution O 1 s spectra (Figure 8c). These O 1 s spectra can be deconvoluted into three peaks, namely, *O*_1_, *O*_2_ and *O*_3_, the binding energies of which are assigned to lattice oxygen in SnO_2_, *O*_latt_ (530.0 eV), oxygen vacancies, *V*_O_ (531.1 eV), and dangling hydroxyl (-OH) groups bounded on the SnO_2_ surface (532.2 eV) [51], respectively. The integral intensities of *O*_1_–*O*_3_ components were calculated from the area under each of corresponding curves, and the ((*O*_2_ + *O*_3_)/*O*_1_), i.e., (*O*_defect_/*O*_latt_) fraction is found to be 0.2112 for the TF(SnO_2_-SG) and 0.3106 for the TF(SnO_2_-R). All data can be found in Table S2. Additionally, the XPS measurement of TF(SnO_2_-R) confirmed the presence of SiO_2_ associated with the preparation process of SnO_2_-R powder. The high-resolution spectrum of Si 2*p* (Figure 8d) showed that Si 2*p*_3/2_ and Si 2*p*_1/2_ lines are located at a binding energy of ≈101.3 and ≈102.0 eV [52], respectively. The calculated fraction of SiO_2_ at the TF(SnO_2_-R) sensor surface was 1.2 at%. Since the WAXS and XRD analyses did not confirm the presence of SiO_2_ in the crystalline forms, it can be concluded that SiO_2_ exists in an amorphous form in both SnO_2_-R powder and TF(SnO_2_-R) sensor, and, according to Gulevich et al. [35], increases the oxygen-related defects in the SnO_2_ film.

In this work, the SiO_2_ domains, remaining after NaOH leaching and partially segregated at the surface of the SnO_2_-R powder, contributed to the inhibition of the SnO_2_ crystal growth and preservation of porous thin-film structure during the thermal step of TF(SnO_2_-R) sensor processing. According to XPS analysis, the segregation of SiO_2_ at the SnO_2_ surface induced an increase in oxygen-related defects, which provided additional adsorption sites for water molecules [23,35]. The high density of surface pores and different composition of surface-active groups compared to TF (SnO_2_-SG) most probably altered the reactivity and charge transport through the TF(SnO_2_-R) sensor, and should be considered as essential for a significant change in the sensor’s resistance and its fast response/recovery speed when it exposed to humid air.

A significant change in impedance response upon the exposure of the TF(SnO_2_-R) sensor to moisture defines its sensing mechanism, which is affected by the physicochemical processes at the sensor surface (Figure S5). In fact, water molecules undergo dissociative adsorption on the TF(SnO_2_-R) surface, and at very low humidity this process obeys the chemisorption mechanism defined by Heiland and Kohl [53] in Equations (1) and (2):

(1) $$H_{2}O_{\left(gas\right)}+Sn_{Sn}+O_{O}↔\left(Sn_{Sn}^{\delta +}-OH^{\delta -}\right)+{\left(OH\right)}_{O}^{+}+e^{-}$$

(2) $$H_{2}O_{\left(gas\right)}+2Sn_{Sn}+O_{O}↔2\left(Sn_{Sn}^{\delta +}-OH^{\delta -}\right)+V_{O}^{++}+2e^{-}$$

Depending on through which of these two mechanisms the adsorption takes place, one or two electrons will be released and injected into the conduction band of the SnO_2_, consequently providing an increase in the concentration of majority free charge carriers, i.e., a decrease in overall resistance. Note that at this stage, the oxygen-related defects detected by XPS analysis at the sensor surface become the preferred sites for the facilitated dissociation of adsorbed water molecules. Since, at the low RH level, only a small number of water molecules are adsorbed at the surface, the sensor impedance will be almost unaffected by either electron donation to the conduction band or even by the proton hopping between adjacent hydroxyl groups that occurs when an electric field is applied [16,54]. Monitoring of these processes was not performed due to the limited operation RH range of our climate chamber when it is set at 25 or 50 °C. If the physisorption occurs within less than one mono-layer, a water molecule attaches to two neighboring, initially chemisorbed, hydroxyl groups through hydrogen bonds. Afterwards, the proton is transferred from the hydroxyl group to the water molecule, forming a hydronium (H_3_O^+^) ion. The protonic migration proceeds through proton transfer from H_3_O^+^ to H_2_O or between neighboring H_2_O molecules in clusters. A further increase in RH leads to multi-layer water adsorption, where each water molecule is single-bonded to a hydroxyl group, and the transport mechanism is assured by H^+^ transfer between adjacent H_2_O molecules within a continuous water film. As the multilayer physical adsorption progresses, the physisorbed water layers gradually exhibit liquid-like behavior, and in such a situation, the charge transport is governed by the Grotthuss chain reaction, causing a significant increase in conductivity, i.e., a decrease in impedance [16,54]. Simultaneously with protonic transport, the electrolytic conduction takes place at a high humidity due to the condensation of the liquid water within the pores (Kelvin’s law) and its rapid propagation towards the electrodes [54,55]. Usually, this leads to the distortion of the low-frequency semicircle and formation of the low-frequency tail [54], which, in our case, is observed in the Cole–Cole spectra at 70 RH% (Figure S5a, see the inset). The proposed mechanism explains a significant impedance reduction (160 times at 25 °C) over the measured RH range (Figure 7d and Figure S5a). Owing to the fact that the number of charge carriers of sensing material and H_3_O^+^ increases (SD, Figure S5a,b) upon the temperature change from 25 to 50 °C, the impedance measured at 42 Hz and 30% RH reduces from 29.0 to 17.3 MΩ, respectively.

The humidity sensing performances of sensors based on highly ordered and mesoporous SnO_2_ materials designed and applied for humidity monitoring over the past decade [16,17,36,37,56,57], and TF(SnO_2_-R) and TF(SnO_2_-SG) sensors processed in this work, are summarized and presented in Table 2. Compared to TF(SnO_2_-SG), the TF(SnO_2_-R) showed a 4.6 times better order of impedance change, 4 and 6.6 times faster response and recovery time, and 3.5 times lower hysteresis error. Compared to others, our sensor exhibits a decent hysteresis error, somewhat lower value of the order of impedance change with a sensitivity of 406.8 kΩ/RH% and a rapid transient response of t_res._⁄(t_rec._ = 4s⁄6s), providing the opportunity to be considered as a promising humidity sensor.

## 3. Materials and Methods

### 3.1. Materials

Reagent grade chemicals were used in hard-templating and sol-gel syntheses: Pluronic F-127 (F-127, (C_3_H_6_O·C_2_H_4_O)_x)_; Mw = 12,600 (Sigma-Aldrich Chemie GmbH, Steinheim, Germany); tetraethoxysilane (Si(OC_2_H_5_)_4_); TEOS, p.a. 98% (Sigma-Aldrich Chemie GmbH, Steinheim, Germany); hydrochloric acid (HCl, ≥37 wt%, Sigma-Aldrich Chemie GmbH, Steinheim, Germany); tin(II) chloride dihydrate (SnCl_2_·2H_2_O, p.a. 98+%, Acros Organics, Geel, Belgium); sodium hydroxide (NaOH, p.a. 98.9%, Lach-Ner, Czech Republic); absolute ethanol (C_2_H_5_OH, Honeywell, Seelze, Germany); and deionized water. The organic functional agents, i.e., α-terpineol (C_10_H_18_O, Sigma-Aldrich Chemie GmbH, Steinheim, Germany), ethyl-cellulose (C_20_H_38_O_11_,Sigma-Aldrich Chemie GmbH, Steinheim, Germany) and glacial acetic acid (C_2_H_4_O_2_, p.a. 99+%, Alfa Aesar, Karlsruhe, Germany) were employed, too.

### 3.2. Syntheses of Mesoporous KIT-5 Template, SnO_2_-KIT-5 Hybrid and SnO_2_ Replica by Hard-Templating

**Figure 9:**
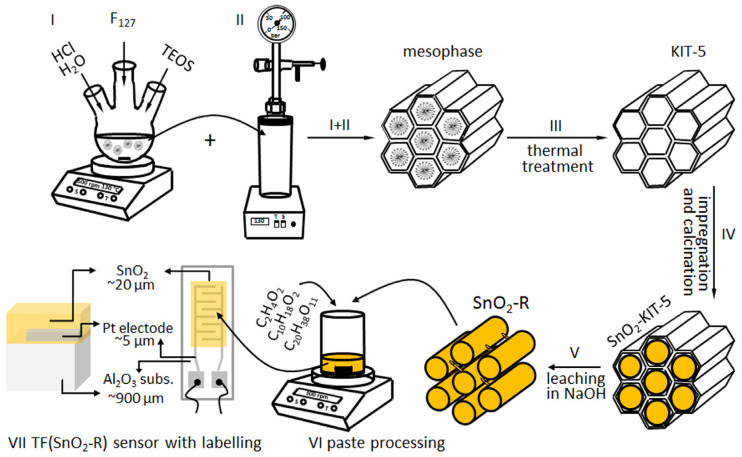
Processing of KIT-5, SnO_2_-KIT-5 and SnO_2_-R powders, paste processing, and TF(SnO_2_-R) top and cross-section illustration with labelling. Note that the SnO_2_-R active layer covers an area of 4 mm × 7 mm at the surface of alumina substrate with interdigitated electrodes.

The synthesis of highly ordered 3D ccp cage-type mesoporous silica KIT-5 was performed in the low acidic media (0.4 M) respecting the molar composition of reaction mixture TEOS: F-127:HCl:H_2_O = 1:0.0035:0.88:119 proposed by Ryoo’s research team [30]. To prepare approximately 100 mL of stock solution, 1.6 g of the tri-block co-polymer F_127_, used as a structure-directing agent, was dissolved in appropriate amount of 0.4 M HCl solution by stirring for 30 min at room temperature. This solution was heated up in the oil bath to reach 45 °C, and afterwards the TEOS (7.6 g) was added to the solution dropwise. The mesophase started to form after 2 h and then stirring was continued at 45 °C for 24 h; Figure 9, step I. Thereafter, the mixture was transferred into teflon-lined autoclave (Carl Roth Model II, Germany) for the hydrothermal treatment at the boarder conditions 130 °C/24 h (Figure 9, step II) according to investigations presented in [30]. The product was filtered, washed with deionized water, dried at 100 °C and homogenized. The mesoporous silica KIT-5 template was obtained after removal of polymer from the above product by calcination in air at 550 °C for 5 h; Figure 9, step III.

To prepare 10 wt% SnO_2_-loaded KIT-5 hybrid (denoted as SnO_2_-KIT-5, the outcome of step IV given in Figure 9) using wet-impregnation method, 1 g of KIT-5 powder was dispersed in SnCl_2_·2H_2_O ethanol solution. The dispersion was agitated to foster the solute species to diffuse into the pores of the matrix, and to stay adsorbed at the pore walls after solvent evaporation step—overnight drying at 50 °C. Two-step calcination of the product in air (300 °C/4 h and 500 °C/6 h; heating rate: 2 °C/min) resulted in the complete thermal decomposition of SnCl_2_·2H_2_O to SnO_2_.

In the next nanocasting step, the SnO_2_-KIT-5 was dispersed in a room-temperature aqueous solution of 1M NaOH, agitated for 2 h and then held for 6 h at 40 °C. Under these mild leaching conditions, KIT-5 template was dissolved, leaving the SnO_2_ replica (denoted as SnO_2_-R) as the final product, which was further washed several times by deionized water, collected by centrifugation (6000 rpm/20 min; Hettich EBA20, Germany) and dried at 50 °C; Figure 9, step V.

### 3.3. Sol-Gel Synthesis of SnO_2_

For comparison, SnO_2_ was synthesized by a sol-gel method from 0.5 M ethanol solution of SnCl_2_·2H_2_O, which was dried overnight at 50 °C to obtain a gel, and further thermally treated at 300 °C/4 h and 500 °C/6 h with a slow heating rate of 2 °C/min (denoted as SnO_2_-SG).

### 3.4. Characterization Methods

The transmission electron microscopy (TEM) was performed using a conventional 200 kV transmission electron microscope (TEM; JEM-2100, Jeol Ltd., Tokyo, Japan) equipped with an energy-dispersive X-ray spectrometer (EDXS). The samples were ultrasonically dispersed in absolute ethanol and drop-casted onto a carbon-coated Ni-TEM mesh. Several SnO_2_ clusters were studied along four- and three-fold axes to reconstruct their mesostructure. To obtain the d spacing of pore channels, TEM images were recorded near Gaussian focus.

Using a high-flux SAXSess camera (Anton Paar, Graz, Austria) with a Debyeflex 3003 X-ray generator (GE-Electric, Germany), operating at 40 kV and 50 mA, with a sealed-tube Cu anode, the small- and wide-angle X-ray scattering (SAXS and WAXS, respectively) measurements were performed on KIT-5, SnO_2_-KIT-5 and SnO_2_-R samples both separately and simultaneously. The Goebel-mirror-focused and Kratky-optic-slit-collimated X-ray beam was used in a line shape (17 mm horizontal dimension at the sample). The SAXS-scattered radiation was measured in the transmission mode and recorded by a one-dimensional MYTHEN-1k micros trip solid-state detector (Dectris Switzerland), within a *q*-range of 0.1 to 6 nm^−^^1^, where *q* was the magnitude of the scattering vector. Using the incident beam of a CuKα radiation (*λ* = 1.54 Å), a sample-to-detector distance of 307 mm, and applying the conversion equation *q*[Å^−^^1^] = 4π(sinθ)/*λ*, the total measured 2θ region was set to be between 0.14 and 7°. In addition, the simultaneous SWAXS measurements were recorded with a 2D imagine plate detector displaying SAXS and WAXS x-scale (*q* or 2θ) without any *q* or 2θ gap in-between. The samples were stored in a rotation-capillary with an external diameter of 2 mm and measured at room temperature applying exposure time of 5 min.

Nitrogen adsorption/desorption isotherms were measured at −196 °C with an ASAP 2020 surface area and porosity analyzer (Micromeritics, USA). Before the measurements, the samples were degassed in a vacuum at 120 °C for 6 h to remove moisture and physisorbed gases. The specific surface area (*S*_BET_) was calculated from the linear part of the adsorption isotherm between 0.05 and 0.3 partial pressure (*P*/*P*_0_) by applying the Brunauer–Emmett–Teller (BET) equation. The mesopore volume (*V*_meso_) and mesopore size distribution with the average pore size value (${\bar{d}}_{\mathrm{meso}}^{\mathrm{BET}}$) were estimated using the Barrett–Joyner–Halenda (BJH) method from the desorption branch of the isotherm.

### 3.5. Fabrication and Testing of SnO_2_ Humidity Sensors

A single-phase SnO_2_-R powder was used to process the functional thick film (TF(SnO_2_-R)), the sensor properties of which were further investigated toward humidity. Powder was mixed with an ethyl-cellulose/α- terpineol = 10/90 wt% solution (S) and acetic acid (A) in the weight ratio SnO_2_-R:S:A = 24:69:7 to form a homogeneous paste (Figure 9, step VI), which was further deposited using the doctor blade technique onto alumina substrate provided with interdigitated Pt electrodes. To avoid mud-like cracking of the film and promote better adhesion between substrate and active layer, the film was submitted to a specific drying/calcination regime consisting of initial drying at 50 °C for 1 h; short intermediate calcination steps at 130, 200, 250, 325, 375, 450 °C for 5 min; and final sintering step at 500 °C for 15 min [58]. The image of alumina substrate with Pt-interdigitated electrodes, contacts, and SnO_2_-R active layer (frontal and cross-section illustrations of the TF(SnO_2_-R) with labelling) is given in Figure 9, step VII. The same procedure was used for the processing of the SnO_2_-SG sensor denoted as TF(SnO_2_-SG).

X-ray photoelectron spectroscopy (XPS) was performed on a SPECS System with an XP50M X-ray excitation source (AlKα radiation, *hν* = 1486.74 eV) for a Focus 500 and PHOIBOS 100 energy analyzer set for recording at 12.5 kV and 16 mA. To follow the chemical states of elements on samples’ surfaces, survey spectra in the binding energy range, 0–1200 eV, were scanned with the pass energy of 40 eV by adopting the energy step of 0.5 eV and dwell time of 0.2 s. The high-resolution XPS spectra of corresponding lines were taken with the pass energy of 20 eV using steps of 0.1 eV and dwell time of 2 s. The adventitious carbon with C 1*s*, C-C line located at 284.8 eV, was used to calibrate samples’ spectra. The data acquisition was performed using SpecsLab data analysis software, while Casa XPS software package was used for further data analysis.

The sensor response of both films towards humidity was tested by measuring the change in the complex impedance by means of a HIOKI 3532-50 LCR HiTESTER in a frequency range 42 to 1 MHz during the sample’s exposure to a humid climate-chamber (JEIO TECH TH-KE-025) environment with the relative humidity, RH, within 30 to 90%; RH at 25 and 50 °C. Note that humid climate chamber, when it is set at 25 and 50 °C, cannot operate below 30% RH. The sensor response was determined as $\mathrm{SR}={\left|Z\right|}_{\mathrm{LH}}/{\left|Z\right|}_{\mathrm{Hi}}$, where ${\left|Z\right|}_{LH}$ is the impedance measured at the lowest analyzed humidity and ${\left|Z\right|}_{\mathrm{Hi}}$ is the impedance measured at the operating RH value. Humidification was performed by inserting the sensor from the external environment, with 37% RH, into the humid chamber previously set to reach the equilibrium at 90% RH. The desiccation process was followed from the starting point of when the sensor was taken out from the chamber (90% RH) into external environment (37% RH), Figure 2 in [59]. The response/recovery time was calculated as the time required by the sensor to reach 90%/10% of its saturation/original value after applying/switching off the certain RH. The reliability of the sensors was discussed by means of the humidity hysteresis error, *HHE*, defined as the time delay between adsorption and desorption processes when the active layer is exposed to a different humidity. It was calculated using the formula: $HHE=\Delta H_{max}/2FSO$, where $\Delta H_{max}$ is the maximum difference in output appearing in adsorption and desorption processes at specified RH value, and *FSO* is the full-scale output.

## 4. Conclusions

The mesoporous SnO_2_-R replica was synthesized through the template inversion of mesoporous silica, KIT-5, to examine the potential application of this material in humidity sensors. In parallel, the conventional sol-gel method was used to synthesize the SnO_2_-SG powder. The thick-film sensors, TF(SnO_2_-R) and TF(SnO_2_-SG), were fabricated from as-synthesized powders. A study on their humidity sensing properties reveals that, unlike TF(SnO_2_-SG), the TF(SnO_2_-R) displays an enhanced humidity sensing response.

1. The sensor shows a relatively low hysteresis error (3.7%), significant change in impedance by 160 times and sensitivity of 406.8 kΩ/RH% over the 30–90% RH range, including a notably fast response (4s) and recovery (6s) time.
2. Such improved humidity sensing properties of TF(SnO_2_-R) arise from the large mesoporous surface area with uniform pore-size, 3D interconnectivity of pore channels and well-crystalized and uniform-in-size SnO_2_ nanoparticles, as shown by TEM.
3. This material provides a high concentration of active sites which creates an effective surface reaction with moisture and facilitates charge propagation across the active-layer surface and mesoporous channels of SnO_2_-R in accordance with the humidity sensing mechanism which facilitates the Grotthuss chain reaction above 60% RH.
4. Contrary to TF(SnO_2_-SG), the presence of amorphous silica at the TF(SnO_2_-R) sensor surface (observed by XPS) contributes to its overall electrical characteristics and creation of additional oxygen related defects. By making the water-molecule dissociation easier, these defects enhance the charge carriers transport across the active-sensing layer and improve sensor performances, including the rapid transient response of the TF(SnO_2_-R) sensor.

The results confirm the advantages of the templating method over the conventional sol-gel method in the processing of a nanosized SnO_2_ powder which meets the criteria of commercialization in the field of humidity sensors.

## Figures and Tables

**Table 1:** Summary of SAXS and BET analyses.

Sample	*R*_g_/nm	Q/nm^−3^	K/nm^−3^	*S*/*V*/m^2^·cm^−3^	${\bar{d}}_{meso}^{BET}$/nm	$S_{BET}/$m^2^·g^−1^
KIT-5	6.6 ± 0.8	53.9·10^−3^	14.9 ·10^−3^	277	6.6	760
SnO_2_-KIT-5	13.6 ± 0.7	2.4·10^−3^	1.7 ·10^−4^	72	6.7	442
SnO_2_-R	14.4 ± 0.6	6.2·10^−3^	9.8 ·10^−4^	158	8.6	66.7

SAXS analysis results: *Rg*—radius of gyration (mean value); Q,—the invariant of the first moment of the smeared intensity; K—Porod constant; *S/V*—surface-to-volume ratio; (more info in SM); BET analysis results: ${\bar{d}}_{\mathrm{meso}}^{\mathrm{BET}}$—average pore size value; $S_{BET}$—specific surface area.

**Table 2:** Humidity sensing performances of the SnO_2_- and SnO_2_-based sensors.

Sample	RH/%	Order of Impedance Change	$t_{res.}$/s	$t_{rec.}$/s	$H_{e}^{25{}^{\circ}C}$/%	Refs
ordered-SnO_2_	11–96	-	32	42	<5	[56]
SnO_2_-SBA15_WI_	11–98	4.5	33	50	2.9	[17]
In-SnO_2_/mesoCN	11–96	5	3.5	1.5	0.7	[16]
SnO_2_ QDs	48–70	-	-	35 *	-	[57]
SnO_2_-graphene	11–98	-	15 **	13 **	5.37	[36]
ZnO-SnO_2_	11–95	4	35	8	6.6	[37]
TF(SnO_2_-SG)	30–90	0.5	16^tw^	40^tw^	13.2	this work
TF(SnO_2_-R)	30–90	2.3	4^tw^	6^tw^	3.7	this work

SnO_2_-SBA15_WI_ – results related to wet impregnation method; *—measured at the fixed RH level and reduced atmospheric pressure; **—measured from 0% to 57% RH and then back to 0% RH, with time intervals of 100 s; tw—tres./trec. were measured from 37% to 90% RH and back to 37% RH.

## Data Availability

The data presented in this study are available on request from the corresponding author.

## Supplementary Materials

The following supporting information can be downloaded at: https://www.mdpi.com/article/10.3390/molecules28041754/s1, Table S1: Comparison between theoretical (JCPDF 01 077-0452) and observed *d*-values of SnO_2_-KIT-5 and SnO_2_-R powders; Table S2: The area under deconvoluted *O*_1_-*O*_3_ and *Otot.* peaks from the high-resolution O 1*s* spectra of different SnO_2_; Figure S1: TEM analysis of KIT-5 pore distribution within SnO_2_-KIT-5: (a) [001] projection, (b) $\left[1\bar{1}0\right]$ projection; Figure S2: EDXS analysis; Figure S3; XRD patterns of SnO_2_-SG and SnO_2_-R powders; Figure S4: (a) The humidity sensing curves of fabricated KIT-5, SnO_2_-KIT-5, SnO_2_-R and SnO_2_-SG sensors presented as the change in complex impedance with a relative humidity change in the 40–90% RH range; (b) the change in the complex impedance with frequency of the TF(SnO_2_-R) sensor at 50 °C exposed to certain RH values; (c) hysteresis behavior of the TF(SnO_2_-R) over 30–90% RH range at 50 °C; Figure S5: Cole–Cole plots of the TF(SnO_2_-R) sensor measured over 30–90% RH range at the temperature 25 °C (a) and 50 °C (b); experimental data are presented using symbols and simulated with lines; Equation (S1): Debye–Scherer equation; Equation (S2): BET equivalent particle diameter; Equations S3–S6: related to SAXS parameters analysis.
