# Supplementary material for: KIT-5-Assisted Synthesis of Mesoporous SnO2 for High-Performance Humidity Sensors with a Swift Response/Recovery Speed
Source: Molecules. 2023 Feb 12;28(4):1754. doi: 10.3390/molecules28041754 (PMC9961371; doi:10.3390/molecules28041754)
Supplement: Supplementary file 1 [file molecules-28-01754-s001.zip › molecules-2149403-supplementary.pdf]

Supplementary Materials – KIT-5-assisted Synthesis of Mesoporous SnO_2 for High-Performance Humidity Sensors with a Swift Response/Recovery Speed

Katarina Vojisavljević [1,*], Slavica M. Savić [2], Milica Počuča-Nešić [1,3], Aden Hodžić [4], Manfred Kriechbaum [5], Vesna Ribić [1,‡], Aleksander Rečnik [6], Jelena Vukašinović [1,3], Goran Branković [1,3,†] and Veljko Djokić [7,8,†]

[1] Department of Materials Science, Institute for Multidisciplinary Research, University of Belgrade, 11030 Belgrade, Serbia
[2] Center for Sensing Technologies, BioSense Institute, University of Novi Sad, 21102 Novi Sad, Serbia
[3] Center of Excellence for Green Technologies, Institute for Multidisciplinary Research, University of Belgrade, 11030 Belgrade, Serbia
[4] Central European Research Infrastructure Consortium, 34149 Basovizza, Italy
[5] Institute of Inorganic Chemistry, Graz University of Technology, 8010 Graz, Austria
[6] Department for Nanostructured Materials, Jožef Stefan Institute, 1000 Ljubljana, Slovenia
[7] Faculty of Technology and Metallurgy, University of Belgrade, 11000 Belgrade, Serbia
[8] Innovation Center of the Faculty of Technology and Metallurgy, University of Belgrade, 11000 Belgrade, Serbia
‡ Present address: Department for Nanostructured Materials, Jožef Stefan Institute, 1000 Ljubljana, Slovenia
† These authors contributed equally to this work.
* Correspondence: KV: kvojisavljevic@imsi.bg.ac.rs;

Transmission electron microscopy

Table S1 Comparison between theoretical (JCPDF 01 077-0452) and observed *d*-values of SnO_2-KIT-5 and SnO_2-R powders.

(hkl)	SnO_2-KIT-5 d_{hkl}/Å	SnO_2-R d_{hkl}/Å	SnO_2 (JCPDF 01 077-0452) d_{hkl}/Å
(110)	3.421	3.374	3.362
(101)	2.715	2.664	2.654
(200)	2.432	2.364	2.378
(111)	2.356	2.325	2.318
(210)	2.170	2.132	2.127
(211)	1.821	1.776	1.771

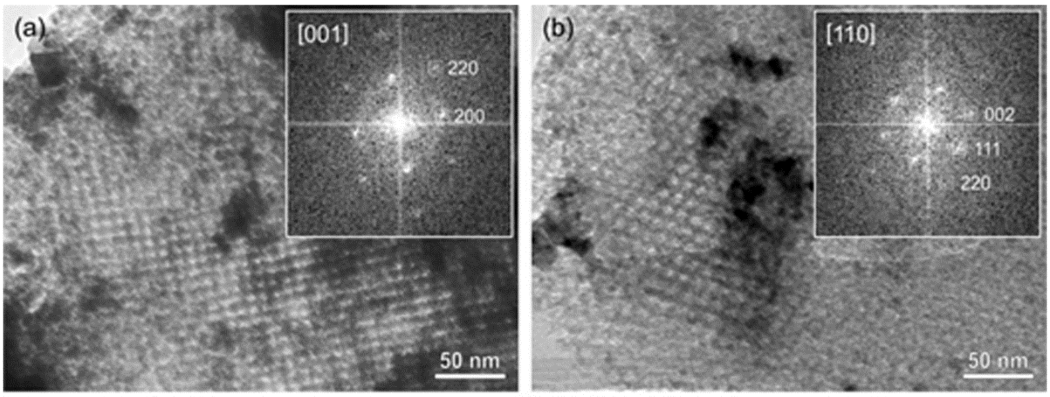

Figure S1. TEM analysis of KIT-5 pore distribution within SnO_2-KIT-5: (a) [001] projection, (b) [$1\bar{1}0$] projection.

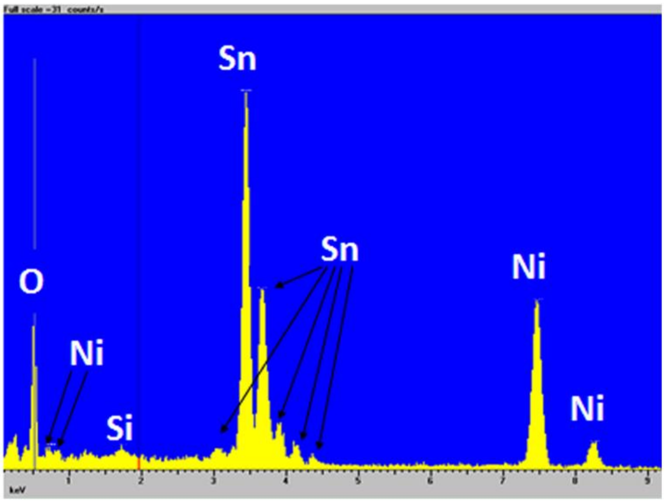

Figure S2. EDXS analysis.

X-ray diffraction analysis

The XRD patterns of SnO_2-R and SnO_2-SG were shown in Figure S3. The average crystallite size of the SnO_2-R and SnO_2-SG particles was calculated by applying the Debye-Scherer equation, Equation (S1):

$$L = K\lambda/\Delta 2\theta cos\theta \quad \text{(S1)}$$

where L is the mean size of the ordered (crystalline) domains, K is a dimensionless shape factor with a value close to unity, λ wavelength (0.154 nm^{-1}), $\Delta 2\theta$ is the line broadening at half of the maximum intensity (FWHM), and θ is the Bragg angle. It appeared to be 10 nm for SnO_2-R and 43 nm for SnO_2-SG.

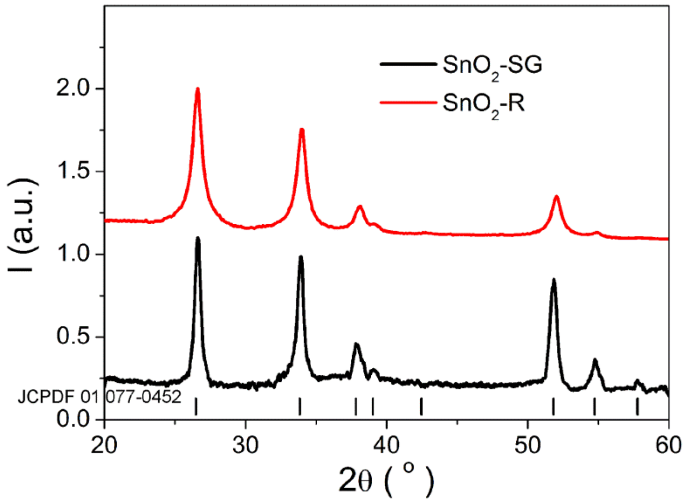

Figure S3. XRD patterns of SnO_2-SG and SnO2-R powders.

BET

Using the S_{BET} values of the SnO_2-R and SnO_2-SG and the formula for the BET equivalent particle diameter, Equation (S2):

$$L_{BET} = (\Psi_A \cdot \Psi_v^{-1})/(S_{BET} \cdot \rho_t) \quad \text{(S2)}$$

where $(\Psi_A \cdot \Psi_v^{-1})$ is the shape factor ratio (=6), S_{BET} is the specific surface area of the powder and ρ_t is the theoretical density of SnO_2 (6.95 g/cm^3) [45], the primary particle size values were calculated to be 12.9 and 42.3 nm for the SnO_2-R and SnO_2-SG, respectively, and they are in agreement with WAXS-XRD/TEM findings.

SAXS Parameters Analytics

The spectra of the same capillary (=the background) were subtracted after merging the spectra and the background spectra at the same transmission line. The radius of gyration R_g and $I(0)$ were determined *via* the Guinier approximation for globular particles (see Equation S3).

$$I(q) = I(0) \cdot exp\left(-q^2 \cdot \frac{R_g}{3}\right) \quad \text{(S3)}$$

The surface S to volume V ration was determined using the approximation derived from Porod's law (see Equation S4) for line-collimated experiments.

$$S/V = 4 \cdot \frac{K_l}{Q_l} \cdot \varphi \cdot (1 - \varphi) \quad \text{(S4)}$$

Here φ is the volume fraction, which was set to $\varphi = 0.5\%$ assuming the general solid state/air ratio for solid samples after transmission normalisation [43,44]. Q_l (the invariant of the first moment of the smeared intensity) was determined by:

$$\mathrm{Q}_l = \int_0^\infty q \cdot I(q)\, dq \quad \text{(S5)}$$

and K_l (the invariant of the first moment of the smeared intensity) was determined by fitting:

$$\mathrm{I(q)} = \mathrm{K}_l/q^3 \quad \text{(S6)}$$

All data were evaluated using SAXS quant 3.100 (Anton Paar GmbH, Austria).

Complex impedance analysis

The change in complex impedance over 30–90% RH range for TF(SnO_2-R) and TF(SnO_2-SG), measured at 50 °C is shown in Figure S4(a). A 1.9 order of change in the impedance magnitude in the 30-90% RH range is observed for the TF(SnO_2-R) sensor, while these values for the and TF(SnO_2-SG) is found to be 0.35 order.

Figure S4(b) presents the frequency dependence of the complex impedance of TF(SnO_2-R) sensor measured in the relative humidity 30–90% RH range at 50 °C. With increase in RH the impedance decreases more slowly with frequency, especially at high RH (above 70% RH). In addition, an increase in RH from 30% to 90% more noticeable influences a decrease of complex impedance in the lower frequency range. For example, at 42 Hz the impedance of 17.3 MΩ is measured at 30% RH and it lowers to 102 kΩ at 90% RH. This operating temperature (50 °C) resulted in similar behavior as observed at 25 °C for TF(SnO_2-R) sensor, but with overall lower measured impedances.

Hysteresis curve is measured at 100 Hz and 50 °C for TF(SnO_2-R) sensor, and it is presented in Figure S4(c). The adsorption and desorption curves for the TF(SnO_2-R) sensor nearly overlay each other showing a small hysteresis error of 3.2%, which represents a good reliability of this sensor.

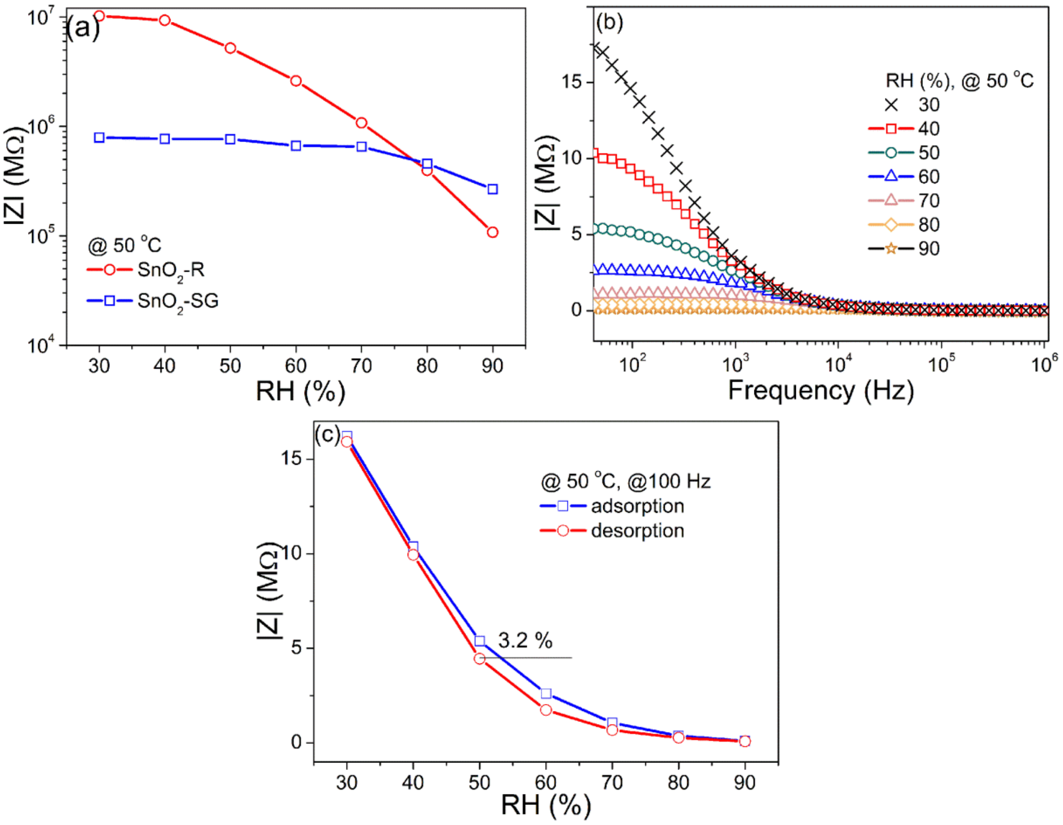

Figure S4. (a) The humidity sensing curves of fabricated, SnO_2-R and SnO_2-SG sensors presented as the change of complex impedance with a relative humidity change in the 30–90% RH range; (b) the change of the complex impedance with frequency of the TF(SnO_2-R) sensor at 50 °C exposed to the certain RH values; (c) hysteresis behavior of the TF(SnO_2-R) over 30-90% RH range at 50 °C.

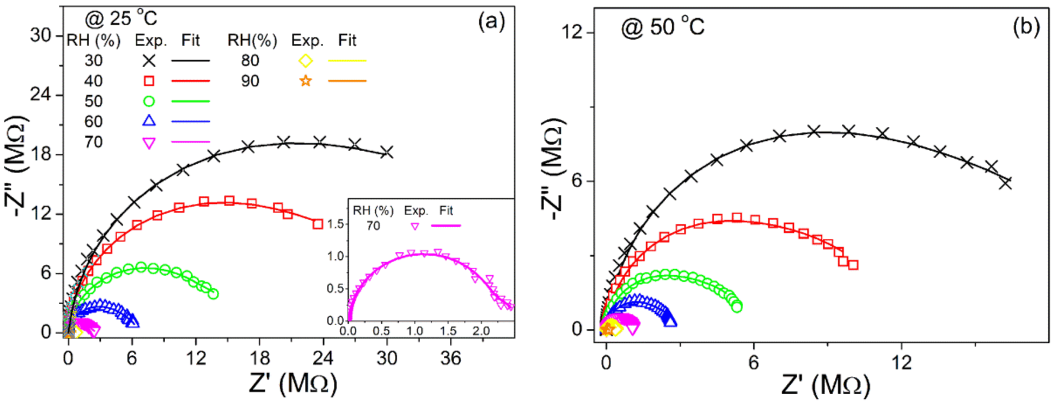

Figure S5. Cole-Cole plots of the TF(SnO_2-R) sensor measured over 30-90% RH range at the temperature (a) 25 °C and (b) 50 °C; experimental data are presented by symbols, simulated by lines.

Table S2 The area under deconvoluted O_1-O_3 and $O_{tot.}$ peaks from the high-resolution O 1*s* spectra of different SnO_2.

Sample	Area under the O_1-O_3 and $O_{tot.}$ peaks						
	O_1	O_2	O_3	$O_{tot.}$	$O_{latt.}$ $O_1/O_{tot.}$	$O_{deff.}$ $[O_2+O_3/O_{tot.}]$	$[O_{deff.}/O_{latt.}]$
TF(SnO_2-SG)	37025	6405	1417	44849	0.8255	0.1744	0.2112
TF(SnO_2-R)	34030	8549	2020	44599	0.7630	0.2370	0.3106

References

[43] A. Hodzic, M. Llusa, N. Heigl, W. Tritthart, S.D. Fraser, P. Laggner, J.G. Khinast, Effect of process variables on the Small and Wide Angle X-ray Scattering (SWAXS) patterns of powders, granules and pharmaceutical tablets, Powder Technol. 221 (2012) 447-452.
[44] A. Hodzic, M. Llusa, S.D. Fraser, O. Scheibelhofer, D.M. Koller, F. Reiter, P. Laggner, J.G. Khinast, Small- and wide-angle X-ray scattering (SWAXS) for quantification of aspirin content in a binary powder mixture, Int. J. Pharmaceut. 428 (2012) 91-95.
[45] J.S. Reed, Principles of Ceramics Processing, John Wiley&Sons, New York, 1995.
